# Analysis of neuropsychological characteristics and related influencing factors in patients with vascular cognitive impairment

**DOI:** 10.12669/pjms.42.5.14949

**Published:** 2026-05

**Authors:** Fengliang Jing, Xin Li, Zhuangwei Xie, Yipan Su, Xiaoyuan Zhang, Chunyu Di

**Affiliations:** 1 Fengliang Jing, Department of Neurology, The Fourth Central Hospital of Baoding, Baoding, Hebei Province 072350, P.R. China; 2 Xin Li, Department of Neurology, The Fourth Central Hospital of Baoding, Baoding, Hebei Province 072350, P.R. China; 3 Zhuangwei Xie, Department of Neurology, The Fourth Central Hospital of Baoding, Baoding, Hebei Province 072350, P.R. China; 4 Yipan Su, Department of Neurology, The Fourth Central Hospital of Baoding, Baoding, Hebei Province 072350, P.R. China; 5 Xiaoyuan Zhang, Department of Neurology, The Fourth Central Hospital of Baoding, Baoding, Hebei Province 072350, P.R. China; 6 Chunyu Di, Department of Neurology, The Fourth Central Hospital of Baoding, Baoding, Hebei Province 072350, P.R. China

**Keywords:** Influencing Factors, Neuropsychological Characteristics, Vascular Cognitive Impairment

## Abstract

**Objectives:**

To explore neuropsychological characteristics and related influencing factors of patients with vascular cognitive impairment (VCI).

**Methodology:**

Clinical data from two hundred VCI patients admitted to the Fourth Central Hospital of Baoding City between May 2021 and October 2023, along with one hundred healthy controls, were retrospectively analyzed. Diagnosis was established based on recognized clinical criteria. Inter-group comparisons of biochemical indices and neuropsychological scores (MoCA/MMSE) were performed using t-tests and chi-square tests, followed by multivariate logistic regression to identify independent associated factors.

**Results:**

Significant differences were observed in lipids, CRP, Hcy levels, and comorbidities (P < 0.05). VCI patients exhibited characteristic deficits in abstract thinking, visuospatial/executive function, and graphical description (P < 0.05). Logistic regression identified low education, alcohol consumption, diabetes, hypertension, and elevated Hcy as independent associated factors (P < 0.05), which specifically correlated with the observed impairments in executive and visuospatial domains.

**Conclusions:**

Neuropsychological characteristics of VCI patients mainly manifest as impairment of abstract thinking, visual spatial and executive function, graphic description, and language expression ability. Lower education level, alcohol consumption, diabetes, hypertension, and high Hcy levels are all important factors significantly associated with an increased risk of VCI.

## INTRODUCTION

Vascular cognitive impairment (VCI) refers to cognitive disorders associated with cerebrovascular disease, and may include vascular dementia, mild cognitive impairment, mixed dementia, etc.^1^,^2^ VCI is considered the second most common type of dementia after the Alzheimer’s disease.^1^–^3^ In recent years, with the gradual aging of the population, the incidence rate of VCI is on the rise, significantly affecting quality of life, and physical and mental health of patients.^4^,^5^

As opposed to Alzheimer’s disease, VCI is preventable and intervenable.^6^ However, if VCI patients do not receive timely and effective intervention, the disease may progress to vascular dementia.^6^,^7^ Therefore, clarifying the influencing factors of VCI and analyzing the neuropsychological characteristics of patients can provide important guidance for development or adjustment of relevant intervention plans in clinical practice that are imperative for improving prognosis.^8^,^9^ This retrospective study aimed to clarify neuropsychological characteristics and related influencing factors of VCI patients.

## METHODOLOGY

Clinical data from two hundred VCI patients admitted to the Fourth Central Hospital Of Baoding City from May 2021 to October 2023 (designated as the VCI group), and one hundred healthy individuals undergoing physical examinations during the same period (normal group) were analyzed. Since this is a retrospective analysis, the sample size was not predetermined by an a priori power calculation. Instead, the study size was naturally dictated by the total number of consecutive cases that fully met the strict inclusion and exclusion criteria within the specified clinical timeframe.

### Ethical approval:

The ethics committee of our hospital approved this study with the number 2024006, Date: January 24, 2024.

### Inclusion criteria for the VCI group:

- The VCI group included patients representing a broad clinical spectrum, ranging from vascular mild cognitive impairment (VaMCI) to vascular dementia (VaD), all of whom met the 2019 Chinese Guidelines for the Diagnosis and Treatment of Vascular Cognitive Impairment.^10^
- Presence of subjective cognitive decline reported by patients or family members. During this phase, functional assessments regarding activities of daily living (ADL) were incorporated via clinical interviews and caregiver feedback to clinically differentiate VaMCI from VaD.
- Objective cognitive impairment confirmed by neuropsychological assessments (MoCA and MMSE).
- Presence of vascular lesions confirmed by MRI (such as multiple infarcts, strategic infarcts, or severe white matter lesions), with a verified temporal or logical correlation between the cerebrovascular event and cognitive decline.
- Age ≥ 18 years old.
- The clinical data is complete.

### Inclusion criteria for the normal group:

- Healthy individuals undergoing routine physical examinations at our hospital during the same period.
- Normal cognitive function confirmed by MoCA and MMSE scores strictly within the clinically recognized normal range, effectively excluding subclinical cognitive impairment.

### Exclusion criteria:

- There is memory impairment, but MRI examination shows no focal damage.
- Patients who undergo long-term interventions aimed to improve cognitive function, or receive sedative drugs, or antidepressants and anxiety medications.
- Patients with Parkinson’s disease, epilepsy, and depression.
- Patients with functional impairment, aphasia, and hearing impairment.
- Patients with cranial tumors.

### Information collected:

### General data:

including gender, age, educational level, smoking and drinking, basic diseases (including heart disease, diabetes, hypertension);

### Biochemical indexes:

Serum levels of low-density lipoprotein cholesterol (LDL-C), high-density lipoprotein cholesterol (HDL-C), triglycerides (TG), total cholesterol (TC) were measured by Roche automatic biochemical analyzer. Levels of C-reactive protein (CRP), and homocysteine (Hcy) were measured by enzyme-linked immunosorbent assay (ELISA). Uric acid (UA) level was measured by fully automatic biochemical analyzer.

### Neuropsychological feature assessment:

Montreal Cognitive Assessment (MoCA) score, and Mini-Mental State Exam (MMSE) score were used. MoCA includes abstract thinking, visual spatial and executive function, language expression, language repetition, object naming, delayed recall, attention and computing power, location orientation, and time orientation; The total MoCA score is 30 points, and higher score indicates better function. MMSE includes graphic description, language expression, reading comprehension, language repetition, object naming, delayed recall, attention and computational power, instantaneous memory, location orientation, and time orientation, with a total of 30 points. Higher score indicates better function.

### Statistical methods:

All data analyses were conducted using SPSS 25.0 software (IBM Corp, Armonk, NY, USA). The measurement data were expressed as mean ± standard deviation, and independent sample t-test was used for inter group comparison. Count data was analyzed using chi square test. To identify the independent associated factors for VCI, a two-stage variable selection strategy was employed. Initially, univariate analyses (independent sample t-tests for continuous data and chi-square tests for categorical data) were conducted to screen all baseline clinical characteristics and biochemical markers. Subsequently, variables demonstrating statistically significant inter-group differences (P < 0.05) in the univariate screening were included as independent variables in a multivariate logistic regression model using the ‘Enter’ method. This approach ensures transparency in model construction and clinical relevance. P<0.05 indicates a statistically significant difference.

## RESULTS

As shown in Table-I, there was no significant difference in gender, age, smoking status, and UA levels between the two groups (*P*>0.05). There were significant differences between the two groups in educational level, alcohol consumption, basic diseases (including heart disease, diabetes, hypertension) and LDL-C, HDL-C, TG, TC, CRP, Hcy levels (*P*<0.05) (Table-I).

**Table-I:** Comparison of baseline data between two groups.

Baseline data		VCI group (n=200)	Normal group (n=100)	χ^2^/t	P
Gender	Male	86 (43.00)	39 (39.00)	0.439	0.508
	Female	114 (57.00)	61 (61.00)	
Age (year)		66.13±7.71	64.92±8.39	1.244	0.215
Smoking	Yes	109 (54.50)	62 (62.00)	1.530	0.216
	No	91 (45.50)	38 (38.00)	
UA (umol/L)		361.12±76.61	358.04±80.66	0.322	0.747
Educational level	Primary school	103 (51.50)	31 (31.00)	11.336	0.001
	Middle and high school	52 (26.00)	11 (11.00)	9.042	0.003
	College degree or above	45 (22.50)	58 (58.00)	37.265	<0.001
Drink	Yes	159 (79.50)	33 (33.00)	62.565	<0.001
	No	41 (20.50)	67 (67.00)		
Heart disease	Yes	59 (29.50)	16 (16.00)	6.480	0.011
	No	141 (70.50)	84 (84.00)		
Diabetes	Yes	69 (34.50)	19 (19.00)	7.727	0.005
	No	131 (65.50)	81 (81.00)		
Hypertension	Yes	83 (41.50)	24 (24.00)	8.898	0.003
	No	117 (58.50)	76 (76.00)	
LDL-C (mmol/L)		3.61±0.71	2.23±0.89	14.549	<0.001
HDL-C (mmol/L)		1.29±0.57	1.94±0.62	9.040	<0.001
TG (mmol/L)		2.78±0.74	2.11±0.59	7.885	<0.001
TC (mmol/L)		5.07±0.95	4.32±1.01	6.311	<0.001
CRP (mg/L)		14.82±2.29	5.44±1.37	37.707	<0.001
Hcy (umol/L)		19.51±5.41	11.67±4.13	12.749	<0.001

***Note:*** UA: Uric acid; LDL-C: Low-density lipoprotein cholesterol; HDL-C: High-density lipoprotein cholesterol; TG: Triglycerides; TC: Total cholesterol; CRP: C-reactive protein; Hcy: Homocysteine.

The scores of abstract thinking, visual space, and executive function in the VCI group were lower than those in the normal group (*P*<0.05). There was no significant difference in the scores for language expression, language repetition, object naming, delayed recall, attention and computational power, location orientation, and time orientation between the two groups (*P*>0.05) (Table-II).

**Table-II:** Comparison of MoCA scores between two groups.

Item	VCI group (n=200)	Normal group (n=100)	t	P
Abstraction	0.79±0.21	1.79±0.38	29.344	0.000
Visual space and executive function	3.22±0.83	4.62±0.79	13.993	0.000
Language expression	0.99±0.21	1.02±0.24	1.111	0.267
Language repetition	1.92±0.34	1.89±0.37	0.699	0.485
Object naming	3.02±0.29	3.04±0.32	0.544	0.587
Delayed recall	3.81±1.10	3.79±1.14	0.147	0.884
Attention and computational power	5.94±0.39	5.97±0.42	0.612	0.541
Location orientation	2.01±0.27	1.99±0.31	0.575	0.566
Time orientation	3.96±0.41	3.98±0.39	0.405	0.686

*Note:* MoCA: Montreal Cognitive Assessment.

The graphic description and language expression scores of the VCI group were lower than those of the normal group (*P*<0.05). There was no significant difference in the scores of reading comprehension, language repetition, object naming, delayed recall, attention and computational power, instantaneous memory, location orientation, and time orientation between the two groups (*P*<0.05) (Table-III).

**Table-III:** Comparison of MMSE scores between two groups.

Item	VCI group (n=200)	Normal group (n=100)	T	P
Graphical description	0.32±0.17	0.81±0.36	16.022	0.000
Language expression	0.59±0.38	1.02±0.27	10.108	0.000
Reading comprehension	3.97±0.51	3.89±0.48	1.306	0.193
Language repetition	0.98±0.18	1.00±0.12	1.005	0.316
Object naming	2.02±0.15	2.01±0.13	0.568	0.570
Delayed recall	2.19±0.64	2.24±0.68	0.625	0.533
Attention and computational power	4.88±0.50	4.92±0.45	0.675	0.500
Immediate memory	2.98±0.28	3.01±0.25	0.906	0.366
Location orientation	4.94±0.36	4.89±0.41	1.082	0.280
Time orientation	4.90±0.44	4.86±0.50	0.709	0.479

***Note:*** MMSE: Mini-Mental State Examination.

Statistically significant indicators were then included in the logistic model for the analysis. Lower education, alcohol consumption, diabetes, hypertension, and increased Hcy levels were all identified as important associated factors for VCI (*P*<0.05) (Table-IV).

**Table-IV:** Analysis of VCI related influencing factors.

Variable	β	S.E.	Waldχ^2^	P	OR	95%CI
Low educational	1.205	0.396	9.261	<0.002	3.337	1.536–7.254
Drink	1.640	0.635	6.673	<0.010	5.157	1.485–17.897
Diabetes	1.540	0.582	7.001	<0.008	4.664	1.491–14.591
Hypertension	1.508	0.531	8.069	<0.005	4.519	1.596–12.794
Increased Hcy	1.267	0.479	6.996	<0.008	3.550	1.388–9.076

***Note:*** OR: Odds ratio; CI: Confidence interval; Hcy: Homocysteine.

## DISCUSSION

This study evaluated the neuropsychological characteristics of VCI patients based on widely utilized clinical screening tools, and showed that both MoCA scores of abstract thinking, visual space, and executive function, and the MMSE scores of graphic description and language expression were significantly lower in the VCI patients compared to healthy population.

Our results indicate that cognitive impairment in VCI patients mainly focuses on abstract thinking, visual spatial and executive functions, as well as graphic descriptions and language expression. These observations are generally consistent with the research results and conclusions of Potocnik J et al.^11^ and Arevalo Rodriguez et al.^12^ Additionally, a study by Kanser et al.^13^ pointed out that cognitive impairment in VCI patients follows a “frontal subcortical” pattern, with early disease mainly characterized by executive dysfunction caused by white matter lesions and cortical lesions. Boomsma et al.^14^ found that VCI patients often exhibit preserved memory but decreased executive ability, which indicates that the frontal lobe structure of the brain is mainly responsible for regulating the body’s executive function, and the completion of executive function mainly depends on the dynamic interaction between the prefrontal cortex and other cortical and subcortical regions. In the context of neuroanatomy, the executive dysfunction observed in our VCI cohort can be further elucidated by the disruption of frontostriatal circuits. Vascular pathologies, such as white matter hyperintensities or lacunar infarcts, often interfere with the white matter tracts connecting the dorsolateral prefrontal cortex (DLPFC) to subcortical nuclei.^15^ As the DLPFC is pivotal for high-level cognitive processes, including abstract thinking, working memory, and executive modulation, the anatomical disconnection within these circuits directly impairs cognitive control.^16^ This provides a clear neuroanatomical basis for the significantly lower scores in abstraction, visual-spatial/executive function, and graphical description recorded in the VCI group compared to the normal group.

Interestingly, our study found no significant inter-group differences in domains such as delayed recall and orientation (P > 0.05). This specific cognitive profile can be elucidated by the underlying neuropathological mechanisms of VCI. Unlike Alzheimer’s disease (AD), which is predominantly driven by early hippocampal atrophy leading to profound episodic memory loss, VCI typically follows a ‘frontal-subcortical’ pattern of ischemic damage.^17^ Vascular lesions, such as white matter hyperintensities, primarily disrupt the prefrontal-subcortical networks. This disruption leads to prominent executive dysfunction and psychomotor slowing, while episodic memory (such as delayed recall) and orientation often remain relatively preserved in the early and mild stages.^18^ Therefore, the relatively intact memory and orientation observed in our cohort align closely with the classic clinical manifestation of early-stage VCI.

In addition, our study explored the influencing factors of VCI. Logistic analysis confirmed that lower education, alcohol consumption, diabetes, hypertension, and increased Hcy levels are all important factors associated with VCI. Notably, our results showed that CRP levels in the VCI group were significantly higher than those in the normal group (14.82 ± 2.29 vs 5.44 ± 1.37 mg/L, P < 0.001). Although CRP was not identified as an independent risk factor in the final logistic model, this significant elevation highlights the pivotal role of chronic low-grade inflammation in the development of VCI. Systemic vascular inflammation, as indicated by elevated CRP, can lead to endothelial dysfunction and promote the formation of atherosclerotic plaques.^19^ Furthermore, persistent inflammatory responses can increase the permeability of the blood-brain barrier (BBB), allowing peripheral inflammatory mediators to infiltrate the brain parenchyma.^20^ This infiltration triggers neuroinflammation, microglial activation, and subsequent neuronal apoptosis, which are closely linked to white matter damage and cognitive decline.^21^ Therefore, targeting vascular inflammation may represent a crucial therapeutic avenue for slowing the progression of cognitive impairment. We may speculate that patients with lower education generally have lower economic income, poor living environment, and delayed access to medical treatment, as well as insufficient mastery and understanding of the scale. Indeed, Jia L et al.^22^ found patients with higher levels of education have stronger computing, memory, and execution abilities, and are able to better adjust to the disease and cooperate with the treatment.

Our study identified alcohol consumption as an associated factor for VCI. Long-term and excessive alcohol consumption may lead to alcoholic encephalopathy, and various deficiencies (such as vitamin B12 and folic acid deficiency), increasing the risk of cerebrovascular disease.^23^ Diabetes was strongly associated with the risk of VCI in our study. Research shows that diabetes can affect large and small blood vessels, causing arteriosclerosis, mitochondrial dysfunction, and subsequent apoptosis of brain cells.^24^ A study by Edgerton-Fulton et al.^25^ showed that increased blood sugar levels can cause systemic microvascular complications and accelerate the decline of body function. Moreover, elevated blood sugar can affect blood pressure and blood lipids, damaging the blood-brain barrier and causing vascular disease and cognitive impairment through oxidative stress.^25^

In our study, hypertension was identified as an associated factor for VCI. Elevated blood pressure can cause contraction of cerebral arterioles, leading to arteriosclerosis and thickening of arteriole walls, as well as proliferation of collagen and elastic fibers in the intima of arterioles over time.^26^ This leads to thickening of the small arterial wall and narrowing of the lumen, resulting in cerebral hypoxia and ischemia, ultimately leading to cognitive decline.^27^ Ungvari et al.^28^ found that long-term hypertension can cause cerebral arteriosclerosis, which in turn affects cerebral blood flow supply, causes nerve cell damage, and increases the risk of VCI.

The increase in the levels of Hcy strongly correlated with the risk of VCI in our study. Overexpression of Hcy can accelerate the process of atherosclerosis, induce apoptosis of hippocampal neurons, and cause VCI.^29^ Song Y et al.^30^ also confirmed that excessive expression of Hcy is an important factor associated with VCI, mainly because Hcy can directly damage nerve endothelial cells and neurons, which is consistent with the viewpoint of this study.

Interestingly, this study did not find a significant statistical association between age and VCI (P = 0.215). Our results contrast with the observations by Shadyab AH et al.^31^, which established age as a primary factor associated with cognitive decline due to age-related organ degradation and reduced dopaminergic neurotransmission efficiency. The unexpected lack of association in our cohort must be interpreted with extreme caution and is likely attributable to specific sample characteristics. First, the average age of our enrolled patients was relatively low (approximately 65 years) with a restricted age span, which may have reduced the statistical power to detect the long-term effects of aging. Second, the retrospective design required complete clinical and neuropsychological data, inevitably introducing selection bias by excluding very elderly or frail patients who could not tolerate comprehensive scale assessments. Finally, the profound impact of vascular and metabolic factors (such as elevated Hcy, diabetes, and hypertension) in our specific cohort may have statistically masked the underlying influence of biological aging. Therefore, this finding is specific to the current cohort and should not negate the universally established role of age in VCI. Further prospective studies with broader age distributions are warranted to comprehensively evaluate this dynamic.

From a clinical perspective, these findings offer actionable insights for screening and intervention strategies. Given the specific cognitive profile of VCI, clinical screening should not rely solely on global cognitive scores. Instead, healthcare providers must prioritize the evaluation of specific sub-domains, particularly abstract thinking in the MoCA and graphical description in the MMSE, to capture subtle frontostriatal deficits at an early stage. Furthermore, intensified cognitive monitoring should be implemented for populations presenting with these associated factors, particularly lower education levels, hypertension, and diabetes. In terms of targeted intervention, the strong association between elevated Hcy and VCI underscores the necessity of incorporating routine Hcy monitoring into clinical management. For patients with hyperhomocysteinemia, early interventions, such as folic acid or B-vitamin supplementation, may be considered to mitigate cognitive decline. Ultimately, a comprehensive secondary prevention strategy—encompassing strict blood pressure and glycemic control, alongside lifestyle modifications such as alcohol cessation—is paramount for delaying the progression from mild vascular cognitive impairment to overt vascular dementia.

### Limitations:

First, the single-center, retrospective design restricts findings to statistical associations rather than definitive causal inferences. While requiring complete clinical data introduces potential selection bias and an a priori sample size calculation was lacking, a post-hoc analysis confirmed robust statistical power (>0.90) to detect clinically meaningful effects. Second, both clinical and structural granularity were limited. We evaluated generalized VCI without severity stratification (VaMCI vs. vascular dementia) and utilized MRI exclusively for qualitative inclusion. The absence of quantitative neuroimaging (such as Fazekas scale, lesion volumetry) restricts our capacity to correlate specific vascular topographies with biochemical or cognitive profiles. Finally, cognitive assessments relied on rapid screening scales (MMSE/MoCA) rather than comprehensive neuropsychological batteries (such as Stroop task). This may introduce ‘ceiling effects’ in domains like memory and orientation, yielding a preliminary screening profile rather than exhaustive cognitive mapping.

## CONCLUSION

The neuropsychological profile of VCI primarily manifests as impairments in abstract thinking, visuospatial/executive function, and graphical description. These deficits are closely associated with independent associated factors, including low education, alcohol consumption, diabetes, hypertension, and elevated Hcy. Early intervention targeting these modifiable factors may improve patient prognosis.

### Recommendations:

Future prospective, longitudinal studies integrating sophisticated cognitive instruments (such as CANTAB) and quantitative neuroimaging are essential to validate these findings and elucidate underlying pathophysiological mechanisms.

### Authors’ contributions:

**FJ:** literature search, study design, and manuscript writing.

**XL, ZX, YS, XZ, and CD:** data collection, analysis, and interpretation. Critical review

**FJ and CD:** Manuscript revision and validation.

**FJ and CD** take full responsibility and are accountable for the accuracy and integrity of all aspects of the work.

All authors have read and approved the final manuscript.
